# Non-thrombotic Pulmonary Embolism Due to Liver Hydatic Cyst: A Case Report

**DOI:** 10.4274/balkanmedj.2016.0391

**Published:** 2017-05-15

**Authors:** Ahmet Şahpaz, Azem İrez, Hatice Gülbeyaz, Mustafa Talip Şener, Ahmet Nezih Kök

**Affiliations:** 1 Department of Histopathology, Erzurum Branch of Council of Forensic Medicine, Erzurum, Turkey; 2 Department of Autopsy, Erzurum Branch of Council of Forensic Medicine, Erzurum, Turkey; 3 Department of Forensic Medicine, Atatürk University School of Medicine, Erzurum, Turkey

**Keywords:** hydatid cyst, Lung, embolism, autopsy

## Abstract

**Background:**

A non-thrombotic pulmonary embolism is defined as embolization to the pulmonary circulation. It may be caused by microorganisms, foreign bodies, different cell types or gas in the pulmonary circulation. Pulmonary hydatid cyst-induced embolization is a rare complication of heart or liver hydatid cysts.

**Case Report:**

We describe the fatal case of a 15-year-old boy without any known prior illness who was admitted to the hospital after feeling unwell and dropping to the ground while playing ball. During the autopsy, a lesional mass, with dimensions of 13x6 cm, was observed in the left lobe of the liver. The histomorphological examination of pulmonary sections showed scolices observed in pulmonary vessel lumina, thus a non-thrombosis hydatid embolism was diagnosed. Based on the findings, the cause of death was recorded as a non-thrombotic hydatid embolism.

**Conclusion:**

The present case is interesting because a non-thrombotic pulmonary embolism rarely results in sudden death, and a definitive diagnosis was possible only by a histopathological examination.

Hydatid disease, or echinococcosis, is a parasitic infection caused by the larval or cystic stage of the intestinal tapeworm *Echinococcus granulosus*. Humans may be infected via the ingestion of eggs. In infected individuals, cysts may develop in the liver or lungs. Echinococcosis is mostly endemic in sheep-raising agricultural areas in the Middle East, South America, Oceania, and along the Mediterranean coast (1). Although the most commonly involved organs are the liver (75%) and lungs (15%), the parasite can be localized in any region of the body (2). Hydatid pulmonary embolism emerges subsequent to the rupture of a hydatid cyst in the right ventricle or atrium, more rarely following the rupture of the cyst wall to the hepatic veins or the inferior vena cava from the liver by haematogenous spread (3,4). According to autopsy findings, embolisms are the result of mechanical obstruction of the blood flow by vesicles and cysts rather than blood clots or additional thrombosis (1,4). The main complications are anaphylactic shock or rupture of the cyst in the pericardium, causing tamponade (5). The most frequent symptom is haemoptysis, but the clinical findings of pulmonary arterial hydatid cyst embolization are not specific (3,4). According to the clinical presentation, hydatic pulmonary embolism cases are classified into three groups: acute fatal cases, subacute pulmonary hypertension cases resulting with death in less than a year, chronic pulmonary hypertension cases (1).

The present case is interesting because a non-thrombotic pulmonary embolism rarely results in sudden death, and a definitive diagnosis was possible only by a histopathological examination.

## CASE PRESENTATION

Written informed consent was obtained from the parent of the patient who involved in this case.

**Figure 1:**
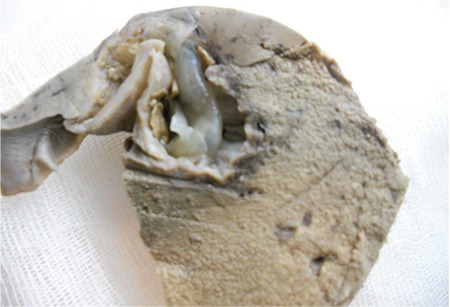
Hydatid cyst in the liver.

**Figure 2:**
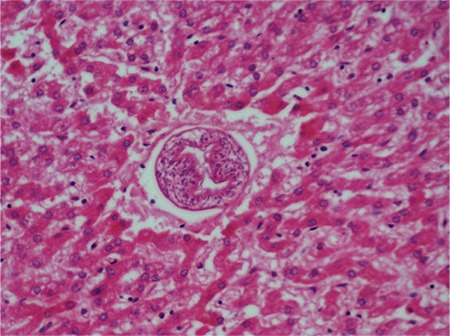
Hydatid embolism in liver (H&E, X200).

**Figure 3:**
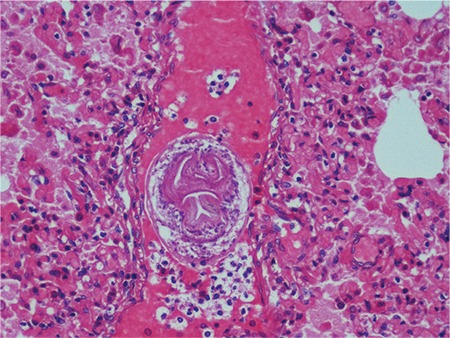
Hydatid embolism in lung (H&E X200).

The case was a 15-year-old boy, without any known prior illness. He was admitted to the hospital after feeling unwell and dropping to the ground while playing ball, and subsequently died. During the internal autopsy examination in our institution, a cystic structure, with dimensions of 13x6 cm, was observed in the left lobe of the liver. Hydatid cyst was not found in other organs of the body. In a macroscopic examination of the liver, a smooth-bordered cystic structure, with dimensions of 13x5x4.2 cm, was observed (Figure 1). On the sectional surface, a white membranous structure and haemorrhagic fluid were observed. The histomorphological examination of liver sections obtained from the cystic area revealed findings consistent with a hydatid cyst invading the external wall, the cuticular layer, and the germinal layer (Figure 2). In addition, the histomorphological examination of liver sections showed a fresh haemorrhage in areas near the cystic wall and scolices in the hepatic lumina (Figure 2), and the histomorphological examination of pulmonary sections showed scolices observed in pulmonary vessel lumina, thus a non-thrombosis hydatid embolism was diagnosed (Figure 3). No toxic agent was identified in a toxicological analysis. Based on the findings, the cause of death was recorded as a non-thrombotic hydatid embolism.

## DISCUSSION

A hydatid cyst is an infectious disease caused by the larval or cystic stage of *E. granulosus*. It is endemic in subtropical and tropical regions of the Mediterranean and South America, Africa and Australia. Infection is transmitted via ingestion of contaminated water or food washed with this water or close contact with dogs (1,2,6). Organisms that reach the gastrointestinal system are carried to the liver via the portal vein and then to the right heart and lungs via the pulmonary artery. They may then remain in the spleen, muscles, and central nervous system or be carried in tissues via the systemic circulation (4,7).

Clinical findings are very variable and depend on the organs involved, the size of the cyst, its location within the organ, and the relationship of the cyst with the biliary tract and vascular structures. They also depend on the complications that develop as a result of cystic rupture, bacterial superinfection of the cyst, and immunological reactions, such as asthma, anaphylaxis, and membranous nephropathy. The first stage of the primary infection is always asymptomatic. Small, well-capsulated or calcified cysts may remain asymptomatic for years or forever if they are not in a location that causes compression. Following an incubation period, which varies among patients, the enlargement of the cyst may lead to symptoms and complications due to compression of adjacent tissues. Usually, these cysts produce symptoms when they reach a certain size. The sudden onset of symptoms may be due to cystic rupture (4,5).

As a diagnostic method, elevation of the right hemidiaphragm, calcifications in pericysts or daughter vesicles (20-30%), pleural effusions or fistulas, and air or fluid may be seen on plain radiographs of the abdomen, depending on the location of the cyst. Ultrasonography is the most important non-invasive diagnostic tool, with specificity of about 90% and sensitivity of 97.7%. Computerized tomography can provide additional information on the location, size, depth, and structure of the cyst and has a sensitivity of 100%. In the diagnosis of hydatid cysts, magnetic resonance imaging is not superior to computerized tomography, but it can provide additional information on the intra- and extrahepatic venous system (1,8).

Hydatid cystic rupture was reported to be a potential cause of sudden unexpected deaths, especially in young adults and children (9). Research also reported that hydatid cystic rupture may be spontaneous in young people who have a history of frequent trauma (10). Despite no known history of trauma in the present case, the diagnosis was spontaneous rupture, as the event developed during sporting activities.

Anaphylaxis, sepsis, multiple organ failure, or fatal embolization of the cystic contents have been reported to be underlying mechanisms of death in cases of hydatid cysts. Arrhythmia, cardiac tamponade, or sudden death due to coronary artery obstruction were also reported to be complications of cardiac hydatid cysts (1,5).

Systemic embolization is frequently associated with cardiac hydatid cyst disease. The rupture of a hydatid cyst and emptying of the cystic contents into the venous or arterial system may lead to a pulmonary or systemic embolism, chronic pulmonary hypertension, and sudden death. A pulmonary embolism is a very rare complication of a liver hydatid cyst (3,6). In the present case, because of the fresh haemorrhage found in areas near the cystic wall and daughter vesicles in hepatic vascular structures, we concluded that the hydatid cyst had ruptured in the vascular structures.

In conclusion, rupture and sudden unexpected death may develop during the follow-up and treatment period of patients clinically diagnosed with a hydatid cyst. If a cystic lesion is encountered in autopsies of cases of sudden deaths of young adults and children, a detailed histopathological examination of the specimens should be conducted to exclude cause of death due to hydatid cysts.
